# Minnesota State Board of Veterinary Medical Examiners

**Published:** 1901-11

**Authors:** 


### STATE BOARDS OF VETERINARY MEDICAL EXAMINERS.

### MICHIGAN STATE BOARD OF VETERINARY MEDICAL EXAMINERS.

### MINNESOTA STATE BOARD OF VETERINARY MEDICAL EXAMINERS.

THE law of Minnesota requires that all residents of the State before beginning the practice of veterinary medicine, surgery, or dentistry shall be graduates of some legally authorized veterinary college or university, and shall pass the examination required by the State Board of Examiners. Anyone who holds a certificate of qualification from another State of five years' standing may, upon establishing a residence, also be entitled to take the examination. The following graduates have passed the Board this year: T. Lambrechts, graduate of the McKillip Veterinary College, 1901; D. M. McDonald, graduate of the Veterinary Department of McGill University, 1891; Harris Dean, graduate of the Ontario Veterinary College, 1887; R. C. Nickerson, graduate of the Western Veterinary College, 1901.

